# Butyrate: A Link between Early Life Nutrition and Gut Microbiome in the Development of Food Allergy

**DOI:** 10.3390/life11050384

**Published:** 2021-04-23

**Authors:** Margherita Di Costanzo, Nicoletta De Paulis, Giacomo Biasucci

**Affiliations:** 1 Pediatrics and Neonatology Unit, Department of Maternal and Child Health, Guglielmo da Saliceto Hospital, 29121 Piacenza, Italy; n.depaulis@ausl.pc.it (N.D.P.); g.biasucci@ausl.pc.it (G.B.); 2 Department of Translational Medical Science—Pediatric Section, University “Federico II”, 80131 Naples, Italy

**Keywords:** short chain fatty acids, immune tolerance, gut dysbiosis, gut microbiota metabolites, cow’s milk allergy, gut homeostasis

## Abstract

Increased prevalence of food allergies in the last thirty years has been attributed to lifestyle changes in Westernized countries. Among the environmental factors, nutritional factors and their interaction with the gut microbiome in early life are thought to have an important role in the observed epidemiological change. The gut microbiome synthesizes bacterial metabolites, which represent a link among gut microbiome, nutrition, and immune system. The main metabolites produced by gut microbiome are short-chain fatty acids (SCFAs). SCFAs have multiple beneficial effects on human health including protective effects in autoimmune and inflammatory diseases. Among SCFAs, butyrate is essential for maintaining gut immune homeostasis and exerts a pivotal role in immune tolerance with strong anti-inflammatory effects in allergic diseases. Recent findings suggest that butyrate takes part in the development of immunological tolerance to food, especially in the first 1000 days of life. Herein, we provide a critical review of the scientific literature on the role of butyrate for prevention and treatment of food allergies with focus on the complex interplay among early life nutrition, gut microbiome, and immune system.

## 1. Introduction

Increased prevalence of food allergies in the last thirty years has been attributed to lifestyle changes in Westernized countries [1]. Among the changed lifestyle factors, nutritional factors and their close interaction with the gut microbiome are thought to play an important role in the observed epidemiological change, especially early in life, when they influence the development of the immune system and oral tolerance to food antigens [2]. The gut immune system continuously communicates with the wide range of microorganisms that colonize the gut and with the different components of foods that are eaten daily [3]. Bacterial metabolites synthesized by the gut microbiome play an important role in the complex interplay between the gut immune system and gut microbiome [4]. Short chain fatty acids (SCFAs) such as acetate, butyrate, and propionate are the most prevalent metabolites produced by gut microbiota. SCFAs, which result from the bacterial fermentation of dietary fibers in the colon, have multiple beneficial effects in autoimmune and inflammatory diseases because of their impact on the immune system [5,6]. Among the SCFAs, butyrate exerts a crucial role in the development of immune oral tolerance with strong anti-inflammatory effects in allergic diseases [7,8,9]. Interestingly, butyrate is the only SCFA produced exclusively by microbial gut fermentation, while other SCFAs are affected by host metabolism [10]. Recent findings support the hypothesis that butyrate might contribute to the development of immune oral tolerance and in the prevention and treatment of food allergies [11,12,13]. This review summarizes what is currently known about the role of butyrate in food allergies, underlining the complex interplay among early life nutrition, gut microbiome, and immune system.

## 2. Gut Dysbiosis in Food Allergy

### 2.1. Evidence from Human Observational Studies

Growing evidence shows that gut dysbiosis, defined as an imbalance in the gut microbial community, has a decisive role in the onset of food allergy. Data from human observational studies suggest that gut dysbiosis precedes the onset of food allergy, with a crucial role for the timing of dysbiosis [14,15]. The first months of life are very important for the future establishment of gut microbiome, and the first weeks after birth are especially considered as a pivotal period in the interaction between the gut microbiome and the immune system [16]. Gut dysbiosis in early life can predispose to the onset of immune-mediated diseases including allergic diseases [16]. In particular, gut microbiome composition in the first six months of life seems to have a central role in the onset of food allergy [17]. Gut dysbiosis can affect not only the onset of food allergy, but also the natural history of the disease, as suggested by the different characteristics of the gut microbiome observed when comparing children who acquire oral tolerance with patients who have persistent forms of food allergies [17]. The role of gut dysbiosis in food allergies even in adulthood has been suggested by a recent study that performed a microbial and metabolomic analysis of human stool samples obtained from pairs of twins, both with food allergy or one twin with food allergy and the other twin without. This analysis revealed a different composition of the gut microbiome and its metabolites even in adulthood among twins with food allergy compared to those without food allergy, and also in the same pair of twins, suggesting that gut microbiome may play a protective role against the onset of food allergy beyond childhood [18]. Unfortunately, studies characterizing the gut microbiome of patients affected by food allergy are still preliminary. A wide range of microorganisms could be implicated with positive or negative influence on tolerogenic mechanisms and no specific bacterial taxa could be associated with the onset of food allergies [14,17,19,20,21,22,23,24,25,26,27]. Most observational human studies have characterized the gut microbiome in children with IgE-mediated food allergy or in children with sensitization to food allergens. Nevertheless, data on the composition and functions of the gut microbiota in non-IgE-mediated food allergies are not yet fully characterized. It is very interesting to note that children with non-IgE-mediated cow’s milk allergy have a gut dysbiosis characterized by a prevalence of *Alistipes* and *Bacteroides* (Bac 12) when compared to healthy subjects, with overlapping signatures with children with IgE-mediated cow’s milk allergy and markedly lower fecal concentrations of SCFA butyrate than healthy subjects [28,29,30].

### 2.2. Evidence from Animal Models of Food Allergy

Strong evidence from animal models has demonstrated the pivotal role of gut microbiome in the acquisition of immune oral tolerance. Indeed, mice given antibiotics as well as *germ free* mice showed a predisposition to the onset of allergy. In particular, *germ free* mice are unable to develop immune oral tolerance to food antigens, maintaining a Th2 immune response [31,32,33]. The reconstitution of the gut microbiome in the first years of life, but not later, can correct this effect. Notably, gut microbiome can also transmit susceptibility to food allergy. In fact, when *germ free* mice were colonized with gut microbiome derived from sensitized susceptible mice (susceptible because of a gain-of-function mutation in the IL-4 receptor), but not from sensitized resistant mice, susceptibility to food allergy was also transferred to the recipient mice [34]. Among the animal models, interesting evidence about the protective role of the gut microbiome against food allergy derives from the “humanized mouse models”. In particular, it has been shown that *germ free* mice colonized with feces of healthy donors are protected from the development of cow’s milk allergy, when sensitized and then exposed to cow’s milk proteins through an oral challenge. In contrast, *germ free* mice colonized with feces from infants with cow’s milk allergy showed severe allergic responses to cow’s milk proteins [35,36].

## 3. Interrelation among Nutrition, Gut Microbiome, and Immune System: The Role of Butyrate

The gut immune system continuously communicates with the wide range of microorganisms that colonize the gut and with the different components of foods that are eaten daily. Butyrate, a gut-microbiome derived metabolite, has received particular attention for its multiple beneficial effects from the gut to the peripheral tissues. In particular, butyrate has emerged since the discovery of its role in driving Tregs differentiation, maintenance of gut homeostasis, and immune oral tolerance [7,8,9,37,38]. It represents an interesting link between early life nutrition and gut microbiome in the onset of allergic diseases including food allergies [39].

### 3.1. Fibers and SCFA Butyrate Production by Gut Microbiome

Diet impacts the gut microbiome, of which composition and function differs depending on the intake of fat, sugar, and fibers. Dietary fibers are made up of non-digestible carbohydrates derived from plant polysaccharides and oligosaccharides, which are resistant to chemical and enzymatic digestion up to the large intestine. These carbohydrates are the main nutritional source for gut bacteria, and their fermentation leads to the production of SCFAs [40]. Although SCFAs can also be derived from dietary proteins and glycoprotein metabolism, carbohydrates represent the main sources [41]. Depending on gut microbiome composition, the amounts and types of SCFAs produced are variable [41]. Gram-positive anaerobic bacteria, which colonize the human colon, can generally produce butyrate. However, it is more correct to consider butyrate-producing bacteria as a functional group, rather than a true phylogenetic group [42]. In gut dysbiosis, butyrate-producing bacteria are reduced, resulting in a reduction in butyrate production.

### 3.2. Reduced Fecal Levels of SCFA Butyrate in Food Allergy: Evidence from Human Observational Studies

In human observational studies, fecal levels of butyrate are different between allergic and non-allergic children in early life [43]. In particular, gut dysbiosis with reduced fecal butyrate levels was found in children with IgE-mediated and non-IgE-mediated cow’s milk allergy compared to healthy controls [21,29]. On the other hand, high fecal butyrate levels in early life are linked to a protective effect against the onset of food allergy [44]. In addition, an enrichment of butyrate-producing bacteria was described in children with faster resolution of cow’s milk allergy, also suggesting a role of butyrate in the history of food allergy [17]. Significant associations were observed between diet composition and fecal levels of SCFAs, thus diet can be an effective tool to modify microbial SCFA production. Roduit et al. [44] analyzed SCFA concentrations in stool samples obtained from 301 one-year-old children and evaluated their association with early life exposures, particularly diet, and allergic manifestations later in their life. They observed that children with the highest stool concentrations of butyrate at one year of age had significantly less atopic sensitization to food and/or inhalant allergens and were less likely to have asthma between three and six years as well as a reported diagnosis of food allergy or allergic rhinitis. Furthermore, Cait et al. analyzed the fecal microbiomes of 105 atopic children at three months and one year of age using shotgun sequencing. They found a positive correlation between butyrate-producing bacteria depletion at three months of age and the development of allergic manifestations later in life. Analyzing the gut microbiome function, the authors also observed that these last infants lacked genes encoding key enzymes for carbohydrate breakdown and butyrate production [10]. These longitudinal studies confirm that early gut butyrate production is protective against the onset of allergic diseases later in life (Table 1). 

### 3.3. Early Life Nutrition and SCFA Butyrate in Food Allergy

**Figure 1:**
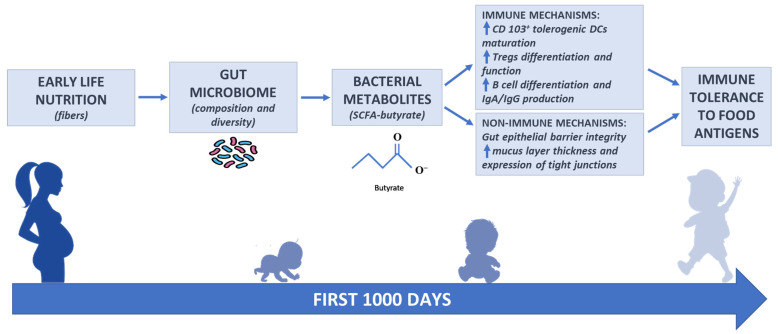
Infant gut microbiome composition and function is related to multiple environmental factors, especially nutritional factors in the first 1000 days of life. These factors are responsible for gut microbiome composition and diversity, related to the different production of bacterial metabolites. The main class of gut microbiome-derived metabolites are SCFAs. Among the SCFAs, butyrate is essential for gut homeostasis with strong anti-inflammatory effects. Butyrate exerts a pivotal role in immune tolerance to food antigens later in life through immune and non-immune mechanisms of action.

Nutrition is a major environmental factor in early life. In this period, nutrition and other environmental factors can influence not only the onset of food allergy, but also the onset of other allergic diseases and immune mediated disorders. These environmental factors act during the first 1000 days of life, a window of opportunity that ranges from pregnancy to the first two years of life. During this vulnerable period, nutritional and other environmental factors strongly influence gut microbiome composition and function (bacterial metabolites) and immune system development (Figure 1). 

During pregnancy and lactation, maternal diet influences not only maternal gut and mammalian gland microbiome, but also infant gut microbiome [45]. Other protective factors against the onset of food allergy are vaginal delivery, breastfeeding, rural environment, increased family size, exposure to pets, a high-fiber diet, and/or fermented food. These environmental factors determine gut eubiosis, characterized by a prevalence of butyrate-producing bacteria and gut barrier integrity, which in turn favor a state of wellness and long-term protection against food allergies in adulthood. In contrast, cesarian delivery, a junk-food-based and/or low-fiber diet during pregnancy and lactation, and exposure of children to antiseptic agents and drugs (especially antibiotics and gastric acidity inhibitors) may lead to a predominant colonization by pathogenic bacteria (gut dysbiosis), reduced production of immunomodulatory factors, and increased gut barrier permeability, with consequent increased risk for the onset of food allergy [45,46,47,48,49,50,51,52].

#### 3.3.1. Breastfeeding: Butyrate as Bioactive Human Milk Protective Component against Food Allergy

The role of breastfeeding in the prevention of food allergies has not been conclusively clarified. The immunological components of human milk would influence the acquisition of immune oral tolerance and decrease the risk of allergic diseases [53,54]. Human milk is a complex biological system and many bioactive factors present in human milk can affect infant immune system maturation [55]. A recent study by Paparo et al. [11] suggests the importance of butyrate as a bioactive human milk component that can protect against the onset of food allergies. Human milk contains a significant level of butyrate. At the detectable concentration in human milk, butyrate can modulate several tolerogenic mechanisms underlying immune oral tolerance to food antigens. In a mouse model, butyrate has been shown to upregulate the expression of several biomarkers of gut barrier integrity as well as tolerogenic cytokines. In animal models of food allergy, pre-treatment with butyrate significantly reduced allergic responses because of an upregulated expression of tolerogenic cytokines, an inhibition of Th2 cytokine production, and a modulation of oxidative stress [11]. In human enterocytes, butyrate has been shown to stimulate mucin production, tight junctions, and human beta defensin-3 expression. In peripheral blood mononuclear cells (PBMCs) from children with food allergy, butyrate enhanced IL-10, IFN-γ, and Forkhead box P3 (*FOXP3)* expression through epigenetic mechanisms. Moreover, it promoted dendritic cells, regulatory T cells (Tregs), and the precursors of M2 macrophages [11]. The authors concluded that an effective concentration of butyrate in human milk could contribute to explain the protective role of breastfeeding against food allergy. Therefore, a valid strategy to increase the protective role of human milk against the onset of food allergies could be based on increasing the concentration of butyrate in human milk through the modulation of maternal diet.

#### 3.3.2. Food Allergy and Relation to Diet and Microbial Metabolites

Grimshaw et al. evaluated the relationship between infant diet composition in the first year of life and the onset of food allergy by two years of age. They concluded that an infant diet consisting of high levels of vegetables, fruit, and home-prepared foods is associated with fewer food allergies by two years of age [56]. Several studies suggest that a good level of adherence to the Mediterranean diet in early life protects against the onset of asthma and atopic manifestations in children [57,58]. Mediterranean diet is composed of high levels of fibers found in fruit, vegetables, legumes and nuts, olive oil, moderate consumption of red wine, poultry and fish, and a lower intake of red meat and sweets. The observed effects could derive from the high intake of non-digestible carbohydrates, whose bacterial fermentation leads the production of SCFAs with a protective role against allergic diseases. Good adherence to the Mediterranean diet has been linked to increased levels of *Prevotella* bacteria, other *Firmicutes*, and SCFAs production [59]. Conversely, the Western diet is characterized by the high consumption of fat and sugar, and low consumption of fibers may contribute to the development of allergic diseases in adolescents and children [60,61]. For example, consuming a high-fat diet can alter the composition of the gut microbiome away from microbes that are efficient at digesting fibers and reduce levels of SCFA butyrate in the colon or blood [62].

#### 3.3.3. Emerging Role of Butyrate in the Active Diet-Therapy in Pediatric Patients with Cow’s Milk Allergy

Cow’s milk allergy is the earliest and the most prevalent form of food allergy in the pediatric age [63]. It can be one of the first manifestations of the so called “atopic march”, characterized by the development of other allergic diseases later in life. The current disease management is based on the elimination diet, access to rescue medication, and use of substitutive formulas [64]. However, dietary management of cow’s milk allergy is shifting from a “passive” elimination diet to an “active diet therapy” that can modify the natural history of food allergy, reducing disease duration, and protecting against the progression of “atopic march” [65]. The latter strategy is supported by a better understanding of the role of early life nutrition, gut microbiome, and tolerogenic mechanisms. It has been demonstrated that in children with cow’s milk allergy, an extensively hydrolyzed casein formula (eHCF) containing *Lactobacillus rhamnosus* GG (LGG) induced higher oral tolerance rates after six and twelve months of treatment compared with eHCF alone and other special formulas commonly used for the treatment of cow’s milk allergy [66,67]. At the three-year follow-up of a pediatric cohort of 220 infants with cow’s milk allergy, a higher rate of oral tolerance acquisition was confirmed after treatment with eHCF plus LGG compared with eHCF alone, and a lower incidence of other allergic manifestations (“atopic march”) was also observed [68]. The use of eHCF supplemented with LGG could exert a modulation of immune tolerance network mediated, at least in part, by the activity of LGG on gut microbiome composition and function, leading to an increased production of the SCFA butyrate. eHCF plus LGG is able to modulate gut microbiome composition and function, increasing the abundance of genera with increased production of SCFA butyrate (such as *Roseburia, Coprococcus*, and *Blautia*) [21]. The efficacy of butyrate was also demonstrated in enhancing desensitization of effector cells induced by oral immunotherapy in a mouse model of cow’s milk allergy. With regard to this, effective reduction of mast cell activation upon in vivo and basophil activation upon ex vivo challenge, and enhanced suppressive activity of oral immunotherapy plus butyrate-derived Tregs have been demonstrated [69].

### 3.4. Butyrate: Immune and Non-Immune Mechanisms of Action Against Food Allergy

**Figure 2:**
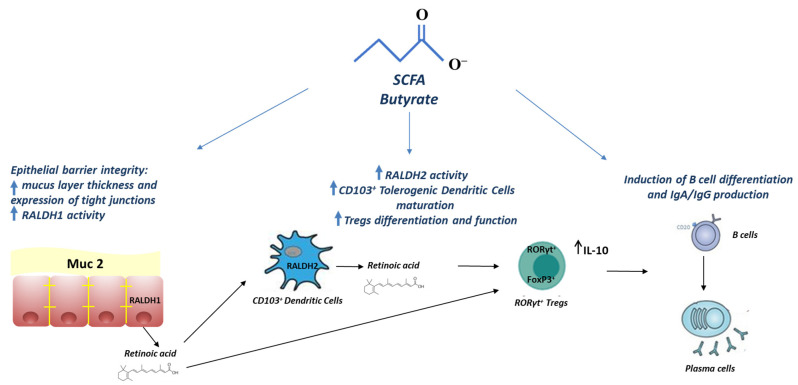
Immune and non-immune protective responses against food allergies elicited by SCFA butyrate at the intestinal level. Abbreviations: Muc 2, mucin 2; RALDH, retinaldehyde dehydrogenases; RORγt, retinoic acid-related orphan receptor γt; FoxP3, Forkhead box P3.

Butyrate stimulates immune and non-immune protective responses against food allergies. It represents the main energy source for colonocytes and influences the expression of genes involved in gut epithelial barrier permeability and defense function. Increased gut epithelial barrier permeability determines increased antigen uptake and promotes Th2-type allergic response by activation of ILC2s, mast cells, basophils, and dendritic cells [70]. Butyrate can improve gut epithelial barrier integrity through increasing the thickness of the mucus layer (enhancing the expression of mucin genes, in particular MUC2) [11,37] and the expression of tight junctions [11,71]. Among the immune mechanisms of action, butyrate acts through different pathways. One of the molecular mechanisms by which SCFAs including butyrate modulate immune system functions is through binding to specific G protein-coupled receptors (GPR) such as GPR43, GPR41, and GPR109a. These receptors are expressed not only on intestinal epithelial cells (IECs), but also on gut immune cells such as Tregs and dendritic cells. Butyrate affects gut CD103^+^ dendritic cells by stimulating the GPR109a cell surface receptor, which allows this tolerogenic dendritic cell subpopulation to trigger proliferation and expansion of Tregs in mesenteric lymph nodes [72]. Furthermore, the GPR109a receptor mediates butyrate induction of IL-18 in colonic epithelium, which is responsible for strengthening the tolerance to commensal bacteria and promoting gut homeostasis. Butyrate is also able to enhance vitamin A metabolism, which in turn induces the activity of aldehyde dehydrogenases (ALDH) in gut CD103^+^ dendritic cells and increases the percentage of Tregs and IgA production [73]. Moreover, high-fiber diet-induced activation of GPR43 and GPR109A activates the NLRP3 inflammasome, which is essential for gut homeostasis [74]. Butyrate can also act through epigenetic mechanisms. It can passively cross the cell membrane, thus inhibiting histone deacetylases (HDAC) in epithelial and gut immune cells. Acetylation/deacetylation of histones is an epigenetic mechanism, which modulates cellular gene expression without modifying genomic DNA sequence. The butyrate-mediated downstream epigenetic effect on enterocytes is expressed by regulation of the expression of genes involved in energy metabolism, cell proliferation and differentiation, and strengthening of the gut barrier integrity through increased expression of tight junctions and enhanced mucus layer thickness [75]. Through HDAC inhibition, butyrate also regulates Tregs size and function in the colon. Indeed, inhibition of HDAC increased *FOXP3* expression and Tregs numbers enhanced the suppressive function of FoxP3^+^ Tregs under homeostatic conditions and amplified Tregs cell-mediated attenuation of colitis in mice [7,8]. Through HDAC inhibition, butyrate can also induce B cell differentiation and production of IgA and IgG [76] (Figure 2).

Furthermore, butyrate stimulates the production of tolerogenic cytokines (IFN-γ and IL-10) in PBMCs [77]. Butyrate induced HDAC3 inhibition promotes macrophage maturation and induces antimicrobial activity in vivo [78]. Overall, immune and non-immune mechanisms of action of SCFA butyrate are essential for gut homeostasis. 

## 4. Conclusions

Current evidence suggests that gut microbiome is influenced by multiple environmental factors including nutritional factors, especially in early life. Gut eubiosis modulates, at least in part through butyrate, gut barrier permeability and enhances a tolerogenic environment by immune mechanisms of action. Starting from immune and non-immune mechanisms of action, butyrate represents an interesting link between early life nutrition and gut microbiome in the development of food allergy. This complex interplay and its role in the pathogenesis of food allergy in early life should be better explored in future studies to lay the foundations for new approaches to prevent or treat food allergies.

## Figures and Tables

**Table 1:** Main evidence from human observational studies on the fecal levels of SCFA butyrate in the first years of life and food allergy/sensitization later in life (↑: increase; ↓: decrease).

Cait et al. 2019 (n = 105)	↓ Fecal levels of SCFA butyrate (stool samples collected at 3 months and 1 year of age)	↑ Food allergy/food sensitization at 1 and 3 years	Ref. [10]
Roduit et al. 2018 (n = 301)	↑ Fecal levels of SCFA butyrate (stool samples collected at 1 year of age)	↓ Food allergy/food sensitization up to 6 years	Ref. [44]
Sandin et al. 2009 (n = 139)	↓ Fecal levels of SCFA butyrate (stool samples collected at 1 and 4 year of age)	↑ Food allergy/food sensitization at 1 and 4 years	Ref. [43]

## Abbreviations

ALDH	Aldehyde dehydrogenases
eHCF	Extensively hydrolyzed casein formula
FoxP3	Forkhead box P3
GPCRs	G-protein coupled receptors
HDAC	Histone deacetylases
IECs	Intestinal epithelial cells
ILC	Innate lymphoid cells
IFNγ	Interferon gamma
IL	Interleukin
LGG	Lactobacillus rhamnosus GG
MUC2	Mucin2
NLRP3	NOD-, LRR-, and pyrin domain-containing protein 3
PBMCs	Peripheral blood mononuclear cells
RALDH	Retinaldehyde dehydrogenases
RORγt	Retinoic acid-related orphan receptor γt
SCFAs	Short-chain fatty acids
Th	T helper
Tregs	Regulatory T cells
